# Fingolimod Rescues Memory and Improves Pathological Hallmarks in the 3xTg-AD Model of Alzheimer’s Disease

**DOI:** 10.1007/s12035-021-02613-5

**Published:** 2022-01-15

**Authors:** Steven G. Fagan, Sibylle Bechet, Kumlesh K. Dev

**Affiliations:** Drug Development, School of Medicine, Trinity College Dublin, Dublin, Ireland

**Keywords:** Alzheimer’s disease, FTY720, Sphingosine-1-phosphate, Inflammation

## Abstract

Therapeutic strategies for Alzheimer’s disease (AD) have largely focused on the regulation of amyloid pathology while those targeting tau pathology, and inflammatory mechanisms are less explored. In this regard, drugs with multimodal and concurrent targeting of A*β*, tau, and inflammatory processes may offer advantages. Here, we investigate one such candidate drug in the triple transgenic 3xTg-AD mouse model of AD, namely the disease-modifying oral neuroimmunomodulatory therapeutic used in patients with multiple sclerosis, called fingolimod. In this study, administration of fingolimod was initiated after behavioral symptoms are known to emerge, at 6 months of age. Treatment continued to 12 months when behavioral tests were performed and thereafter histological and biochemical analysis was conducted on postmortem tissue. The results demonstrate that fingolimod reverses deficits in spatial working memory at 8 and 12 months of age as measured by novel object location and Morris water maze tests. Inflammation in the brain is alleviated as demonstrated by reduced Iba1-positive and CD3-positive cell number, less ramified microglial morphology, and improved cytokine profile. Finally, treatment with fingolimod was shown to reduce phosphorylated tau and APP levels in the hippocampus and cortex. These results highlight the potential of fingolimod as a multimodal therapeutic for the treatment of AD.

**Supplementary Information:**

The online version contains supplementary material available at 10.1007/s12035-021-02613-5.

## Introduction

Alzheimer’s disease (AD) is an age-related neurodegenerative condition and the most common form of dementia which affects approximately 44 million people worldwide [55]. Symptoms of AD include the progressive loss of memory and cognition resulting in a dependency on full-time care [4]. Current treatments, including cholinesterase inhibitors and N-methyl-D-aspartate receptor antagonists, aim to improve symptom severity but do not affect the course of the disease [76]. With an increasingly aged global population and ongoing failures in drug discovery efforts, the burden of AD and other dementias are a serious concern for healthcare systems worldwide [49, 55].

The characteristic hallmarks of AD include the development of extracellular amyloid plaques and intracellular neurofibrillary tangles [21]. Amyloid plaques form through the aggregation of amyloid-*β* (A*β*), a cleaved product of the amyloid precursor protein (APP) [71]. Mutations to genes encoding APP and the enzymes that cleave APP have been implicated in a small number of AD cases and are regularly used as the basis for animal models [73]. Neurofibrillary tangles are intracellular aggregates of hyperphosphorylated tau. Under pathological conditions tau becomes phosphorylated (phospho-tau), insoluble and aggregates, forming tangles [73]. Mutations to the MAPT gene that encodes tau have been implicated in the development of dementia and increased phosphorylation at positions Ser-396, Ser-400, Thr-403, and Ser-404 [1].

Inflammation in the central nervous system (CNS) has emerged as a key driver in the development of AD [33]. Postmortem analyses have determined a significant upregulation in activated microglia and inflammatory markers in the brains of AD patients, and recent imaging studies have used microglial activation in combination with tau localization as a measure of cognitive decline [46–48, 70]. Previous epidemiological data indicated a reduced risk of developing AD among people receiving NSAID treatment, however, more recent studies have demonstrated that anti-inflammatory drugs alone are insufficient [68, 79]. Increasing evidence has implicated the peripheral immune response in AD pathology where T cell responses specific to A*β* have been observed in blood from AD patients, as has T-cell infiltration in the brains of AD patients where they surround A*β* and tau pathology [51, 52].

Given current failures in therapies for AD, approaches with multimodal targeting of inflammatory and neuronal systems may be worthy of consideration. One such candidate drug is fingolimod (FTY720, Gilenya®), the disease-modifying oral neuroimmunomodulatory therapeutic used in multiple sclerosis and that has been investigated in a number of neurodegenerative diseases [57]. Fingolimod readily passes the blood–brain barrier and when phosphorylated binds to sphingosine-1-phosphate (S1P) receptors (S1P_1–5_), except for S1P_2_ [10, 50]. S1P receptors are G-protein coupled and expressed in many cell types including the immune system and central nervous system. The canonical therapeutic activity of fingolimod arises from its retention of T cells in lymphoid organs, where it binds S1P_1_ on T cells causing receptor internalization thereby inhibiting the S1P-mediated egress of T cells into the blood [9]. We and others have now shown that fingolimod can target S1P receptors expressed on neuronal and glial cells, where they can regulate neurogenesis, neurite outgrowth, myelination, inflammation, astrogliosis, and migration [22, 31, 54, 56–58, 60, 63, 65, 66].

In AD, the S1P system is altered with a reduction in sphingosine kinase-1 and -2, and an increase in sphingosine lyase associated with advancing Braak stages [15, 20, 38]. Furthermore, a progressive decline in S1P is also observed throughout the course of the disease [20]. Previous studies have shown that fingolimod can reduce the production of A*β*, *ϒ*-secretase activity, and the formation of amyloid plaques in vitro and in vivo [7, 72]. Furthermore, pre-symptomatic administration of fingolimod has been shown to prevent cognitive decline and reduce the severity of CNS inflammation [7, 12].

For this study, we utilized the 3xTg-AD mouse model of AD which displays cognitive impairment from 3 months, develops amyloid plaques and CNS inflammation from 6 months, and neurofibrillary tangles of phospho-tau emerge from 12 months of age. Oral administration of fingolimod began after the onset of symptoms, at 6 months, and continued until 12 months of age. Previous studies have demonstrated the preventative effects of fingolimod in models of AD; this study indicates that the beneficial effects of fingolimod are also apparent in older mice with developed pathologies. Treatment with fingolimod rescues memory impairment and reduces inflammation in the CNS. Furthermore, we have demonstrated for the first time that fingolimod reduces tau phosphorylation and APP expression in 3xTg-AD mice.

## Methods

### Animals

Male B6;129-Tg(APPSwe, tauP301L)1Lfa Psen1^tm1Mpm^ mice (3xTg-AD; stock 004,807) and B6129SF2 wild-type controls (WT; stock 101,045) were purchased from Jackson Laboratories. An increasing body of work has demonstrated greater A*β* pathology in female 3xTg-AD mice [27, 30, 53] and conflicting reports exist on sexual dimorphic tau burden [30, 35, 53, 77]. No difference in working memory has been found between male and female 3xTg-AD mice [19], and male 3xTg-AD have been shown to perform worse at spatial memory tasks [69]. Furthermore, a more pronounced alteration in the immune system has been reported in male 3xTg-AD mice [5, 29, 39]. Given the immunomodulatory mechanisms of fingolimod and inflammation being of primary focus to this study male, 3xTg-AD mice were chosen for this study. Mice were age-matched and housed under standard conditions (22 ± 2 °C, 12:12 h light:dark cycle, food and water ad libitum). Experiments were carried out under institutional and governmental guidelines.

### Treatment

From 6–12 months of age fingolimod (Caymen Chemical #10,006,292 1 mg/kg/day) was administered via the drinking water. At 8 and 12 months, blood was drawn from the submandibular vein for lymphocyte count (Sysmex KX-21 blood analyzer) and fluorescent activated cell sorting (FACS) analysis. At 12 months animals were sacrificed by CO_2_ asphyxiation, perfused with phosphate-buffered saline, and one hemisphere was fixed in 4% paraformaldehyde for 24 h for immunohistochemistry. From the remaining hemisphere, the cortex and hippocampus were dissected and snap frozen for biochemical analysis.

### Novel Object Location Test (NOL)

**Fig. 1:**
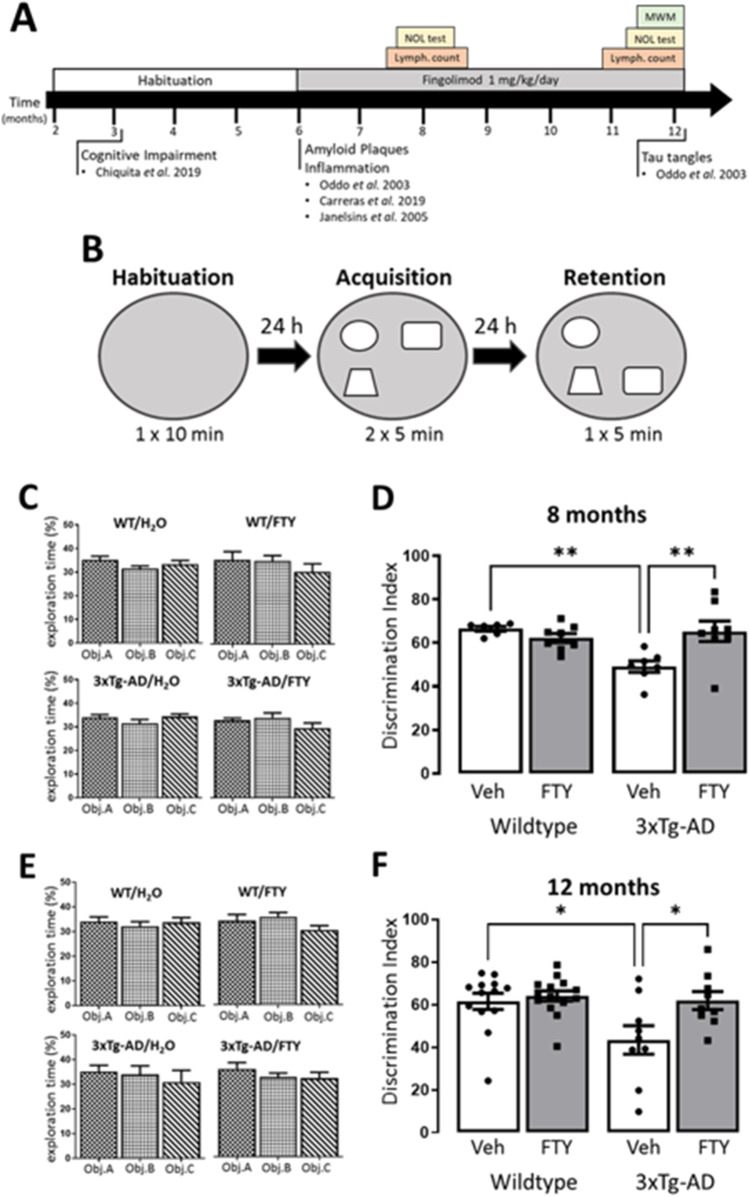
Fingolimod improves performance in the NOL at 8 and 12 months of age. **A** Timeline of reported 3xTg-AD mouse pathology and experimental plan for this study. **B** Experimental design for the NOL test. **C, E** At 8 and 12 months of age mice demonstrated no preference to any of the three objects during the acquisition phase of the NOL test. **D** At 8 months of age a genotype-related reduction in discrimination index was observed (*p* = 0.0332, *F*_(1,25)_ = 5.083). Post hoc analysis revealed that vehicle-treated 3xTg-AD mice performed significantly worse than WT (*p* = 0.0055, *n* = 6–8) and that this was attenuated by treatment with fingolimod (*p* = 0.0054, *n* = 6–8). (**F**) At 12 months of age a genotype-related reduction in discrimination index was observed (*p* = 0.0204, *F*_(1,41)_ = 5.823). Post hoc analysis revealed that vehicle-treated 3xTg-AD mice performed significantly worse than WT (*p* = 0.0211, *n* = 9–14) and that this was attenuated by treatment with fingolimod (*p* = 0.0341, *n* = 9–14). All data was normally distributed and expressed as mean ± SEM, *p* < 0.05, **p < 0.01

The NOL was carried out on a pilot cohort (*n* = 9) at 8 months of age and the full cohort (*n* = 18) at 12 months of age. A cylindrical test arena (50 cm diameter × 30 cm height) with spatial cues and three different objects of similar size were used, and mice displayed no object preference during acquisition trials (Fig. 1C, E). On day 1, mice were habituated (1 × 10 min trial) to the test arena. The next day, mice received acquisition trials (2 × 5 min; 6 min inter-trial interval) and were allowed to freely explore the test arena and the three objects therein. After 24 h, mice were reintroduced to the test arena for a single retention trial (5 min) with one object having moved location (Fig. 1B). Exploration was defined as touching the object with its nose or whiskers, or the animals’ snout was directed at the object at a distance of ≤ 2 cm. Exploration was assessed live by human observations and confirmed through video analysis by blinded analyzers. The results from the live measurements were used here. The discrimination index was calculated as the time spent exploring the displaced object (O_d_) over the average time exploring the stationary objects (O_s_) plus time exploring displaced object multiplied by 100, (O_d_/(O_s_ + O_d_))*100.

### Morris Water Maze (MWM)

**Fig. 2:**
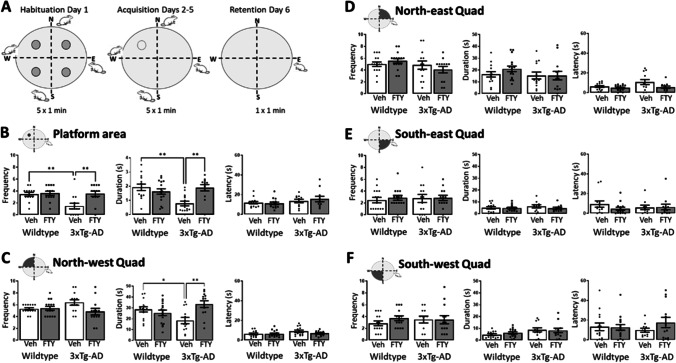
Fingolimod improves MWM performance in 3xTg-AD mice at 12 months of age. **A** Experimental design for the MWM test. **B** A genotype-related reduction in frequency of entries to (*p* = 0.0195, *F*_(1,48)_ = 6.813), and duration in (*p* = 0.0331, *F*_(1,52)_ = 4.79) the platform area, with no difference observed in latency. Post hoc analysis revealed that vehicle treated 3xTg-AD mice entered the platform area less frequently (*p* = 0.0046, *n* = 13) and remained there for less time (*p* = 0.001, *n* = 14) than WT controls. Fingolimod treated 3xTg-AD mice entered the platform area more frequently (*p* = 0.0031, *n* = 13) and for a longer duration (*p* = 0.025, *n* = 12–14) than vehicle treated 3xTg-AD mice. **C** In the north-west quadrant, where the platform was located, a genotype-treatment interaction in duration was observed (*p* = 0.0012, *F*_(1,53)_ = 11.65) but no difference in frequency of entries or latency was noted. Post hoc analysis revealed that vehicle-treated 3xTg-AD mice spent significantly less time in the northwest quadrant compared to WT controls (*p* = 0.0471, *n* = 13–15) and that this was alleviated by treatment with fingolimod (*p* = 0.002, *n* = 13). **D, E, F** No significant difference was observed between groups in frequency of entry, duration in the quadrant, or latency to the quadrant in the north-east, south-east, or south-west quadrants. All data was normally distributed and expressed as the mean ± SEM, *p < 0.05, **p < 0.01

The MWM (145 cm diameter × 60 cm height) was set up with spatial cues surrounding the tank. On day 1, mice carried out 5 × 1 min trials (20 min inter-trial interval) with randomized and changing entry points and a small escape platform (9 cm width × 9 cm length) visible 1.5 cm over the water placed in alternating quadrants. If unsuccessful, mice were guided to the platform and left for 20 s. On acquisition days 2–5, mice carried out 5 × 1 min trials (20 min inter-trial interval) with randomized and changing entry points and the platform location fixed and submerged. If unsuccessful, mice were guided to the platform for 20 s. On day 6, the probe trial consisted of a single 1 min trial with no platform present (Fig. 2A). Swimming traces are included in Sup. Figure 2. The time spent in the platform area, defined as the exact dimensions of where the platform was located in the acquisition trials, was recorded using EthoVision software as a measure of spatial learning and memory.

### Sample Preparation for FACS

Preparation of FACS tissue was carried out as previously described [62]. Cortex from perfused brains was mechanically homogenized and enzymatically digested before straining and separating through a Percoll gradient. Antibodies for CD3 (APC hamster anti-mouse CD3; 1:200; BD Bioscience #553,066), CD11b (PE rat anti-CD11b; 1:200; BD Bioscience #557,397), and CD45 (FITC rat anti-mouse CD45; 1:100; BD Bioscience #553,079) were added to cell suspensions and whole blood samples before the samples were washed and centrifuged, and the pellet resuspended in flow cytometry buffer. Samples were sorted on a FACS Aria™ Fusion cell sorter (Becton Dickenson), lymphocytes were identified as CD3 + , and macrophages were identified as being CD11b + CD45hi.

### Immunohistochemistry

Free-floating 30 µm sections were blocked with 10% BSA, 0.5% triton-X for 2 h followed by incubation in rabbit anti-Iba1 (1:1000; Wako) overnight at 4 °C. Sections were incubated in goat-anti-rabbit ALEXA Fluor-488 (1:1000; Invitrogen #a11008) for 2 h before being counterstained with Hoechst and mounted using SlowFade Gold mounting medium. Z-stack images were taken on a Leica SP8 confocal microscope at × 20 magnification. To measure microglial number Iba1-positive cells were manually counted. Microglial branching was assessed by skeleton analysis as described [78].

### Enzyme-Linked Immunosorbent Assay

IL-6 (DY406) and IL-10 (DY417; R&D Systems) DuoSet ELISA kits were used for cytokine quantification and were carried out as per the manufacturer’s instructions. The plates were incubated in capture antibody overnight at room temperature and then blocked-in reagent diluent. Samples and standards were added, and the plates were incubated overnight at 4 °C. A detection antibody was added for 2 h at room temperature before streptavidin-HPR incubation for 20 min and substrate incubation for 20 min. The reaction was terminated with a stop solution and the plate read at 490 nm.

### Immunoblot

Tissue was manually homogenized (10% w/v) in RIPA buffer containing cOmplete™ mini protease inhibitors (Sigma #04,693,159,001). The homogenate was sonicated (6 × 5 s, 20% pulse), centrifuged (12,000 g; 20 min; 4 °C) and the supernatant stored at − 80 °C. Western blotting was conducted as previously described (Rutkowska et al. 2017) and where required membranes were stripped with ReBlot plus (Millipore) for 3 min. For tau and p-tau, membranes were cut to allow for the simultaneous detection of actin. The primary antibodies were: mouse anti-phospho-tau AT8 (1:500; Thermo Scientific #MN1020), mouse anti-tau46 (1:1000; Cell Signaling #4019), mouse anti-*β*-amyloid 6E10 (1:500; Biolegend #SIG39320), and rabbit anti-*β*-actin (1:1000; Sigma #28,227). The secondary antibodies were: anti-mouse-HRP (1:1000; Sigma #a8924) and anti-rabbit-HRP (1:2000; GE Health #na934).

### Statistics

All statistics were carried out on GraphPad Prism 9 software. The data were normally distributed and data points greater than two standard deviations from the mean were excluded from groups. In all cases, 2-way ANOVA was applied to compare the means and where significance was observed Tukey post hoc correction for multiple comparisons was carried out with *p* < 0.05 as the minimum level of significance. Graphical data is represented as mean ± SEM and post hoc significance indicated as follows: *p < 0.05, **p < 0.01, ***p < 0.001, ****p < 0.0001.

## Results

### Fingolimod Improves Memory Deficits in 3xTg-AD Mice

Cognitive impairment, such as memory loss, is one of the primary symptoms of AD and is known to develop in 3xTg-AD mice from 3 months of age [8, 16]. We assessed spatial learning and memory in 3xTg-AD mice at 8 and 12 months of age, after 2 and 6 months of fingolimod treatment (1 mg/kg/day), respectively (Fig. 1A). At 8 months of age a genotype-related impairment in working memory was observed during the NOL test (*p* = 0.0332, *F*_(1,25)_ = 5.083; Fig. 1D) and post hoc analysis revealed that treatment with fingolimod significantly improved NOL performance in 3xTg-AD mice (Veh: 49.12 ± 2.588, FTY: 65.32 ± 4.7; *p* = 0.0054, *n* = 6–8). The genotype-related impairment was again observed at 12 months of age (*p* = 0.0204, *F*_(1,41)_ = 5.823; Fig. 1F) and post hoc analysis determined that 3xTg-AD mice treated with fingolimod performed significantly better than vehicle-treated controls (Veh: 43.45 ± 6.66, FTY: 61.9 ± 4.224; *p* = 0.0341, *n* = 9–14).

At 12 months of age, MWM was also carried out. No difference in athletic ability was observed as indicated by distance traveled and velocity between the groups (Sup. Figure 1A, B). Escape latency during the training days 2–5 reduced in all groups over time but by training day 5 vehicle-treated 3xTg-AD mice performed significantly worse than their WT counterparts (*p* = 0.017, *n* = 14–18; Sup. Figure 1C). On test day 6, frequency of entry to the platform area (*p* = 0.0195, *F*_(1,48)_ = 6.813; Fig. 2B), duration of time in the platform area (*p* = 0.0331, *F*_(1,52)_ = 4.79; Fig. 2B), and duration of time in the north-west (platform containing) quadrant (*p* = 0.0012, *F*_(1,53)_ = 11.65; Fig. 2C) were all reduced in 3xTg-AD mice compared with WT controls. Post hoc analysis revealed that 3xTg-AD mice receiving fingolimod entered that platform area more frequently than vehicle treated controls (Veh: 1.46 ± 0.48, FTY: 3.54 ± 0.43; *p* = 0.0031, *n* = 13) and spent a longer duration of time in the platform area (Veh: 0.78 ± 0.17, FTY: 1.89 ± 0.22; *p* = 0.0025, *n* = 12–14) and north-west quadrant (Veh: 18.31 ± 2.89, FTY: 33.44 ± 3.07; *p* = 0.002, *n* = 13). Taken together these data demonstrate that fingolimod improves working memory in 8- and 12-month-old 3xTg-AD mice after 2 and 6 months of treatment, respectively.

### Fingolimod Reduces Microglial Activation in 3xTg-AD Mice

**Fig. 3:**
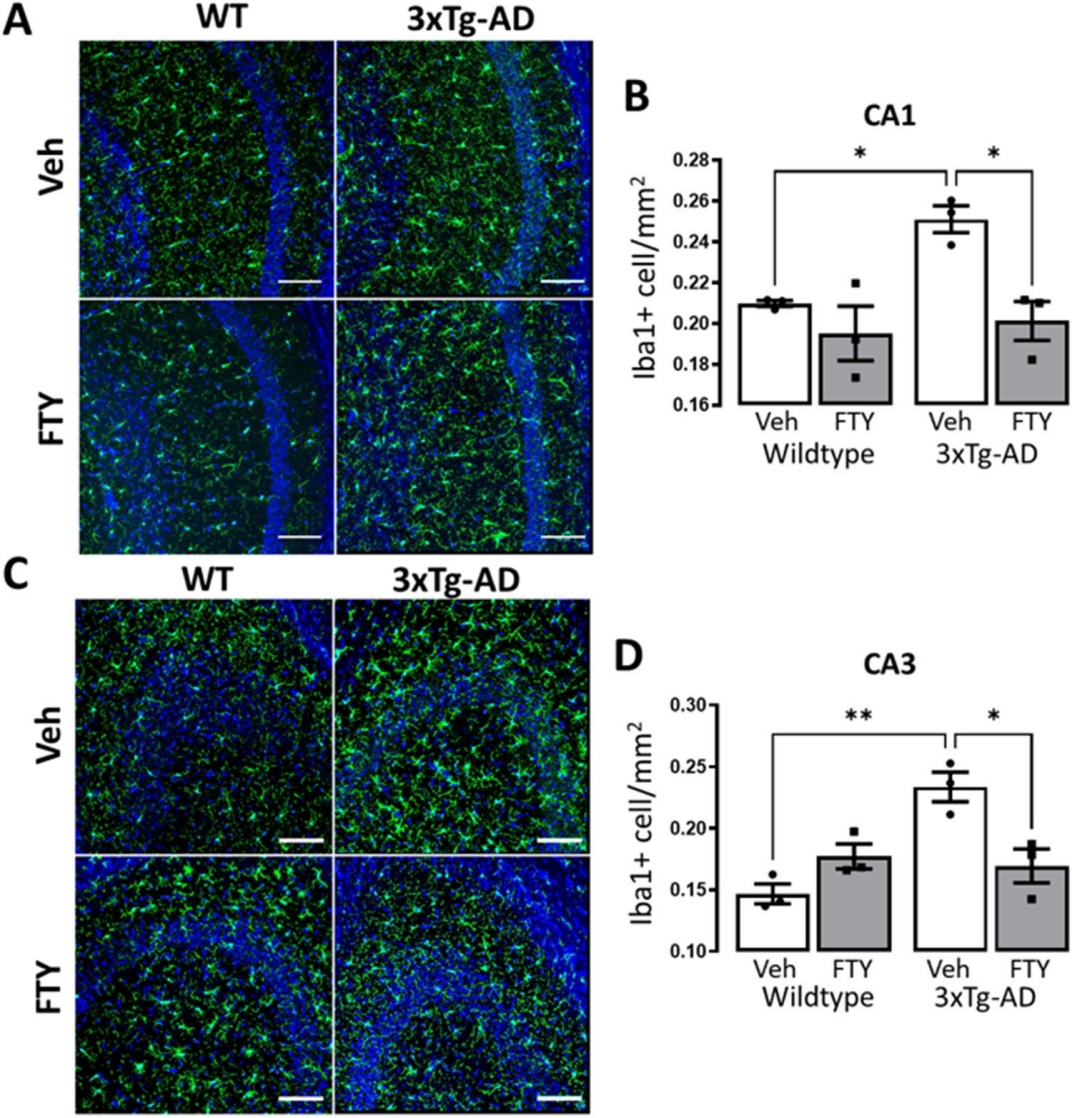
Fingolimod reduces microglial number in 3xTg-AD hippocampus. Iba1 stained sections were used to assess microglial proliferation in the (**A**) CA1 and (**C**) CA3 regions of the hippocampus. A significant genotype-related increase in Iba1-positive cells was observed in the CA1 (**B**: *p* = 0.0286, *F*_(1,8)_ = 7.098) and CA3 (**D**: *p* < 0.0076, *F*_(1,8)_ = 12.56) of 3xTg-AD mice. Post hoc analysis revealed that 3xTg-AD mice had significantly more Iba1-positive cells than WT (**B:** CA1, *p* = 0.0446, *n* = 3; **D:** CA3, *p* = 0.0025, *n* = 3) and that this was alleviated in fingolimod treated 3xTg-AD mice (**B:** CA1, *p* = 0.0174, *n* = 3; **D:** CA3, *p* = 0.0151, *n* = 3). Data are expressed as the mean ± SEM, *p < 0.05, **p < 0.01. Scale bar 100 µm

**Fig. 4:**
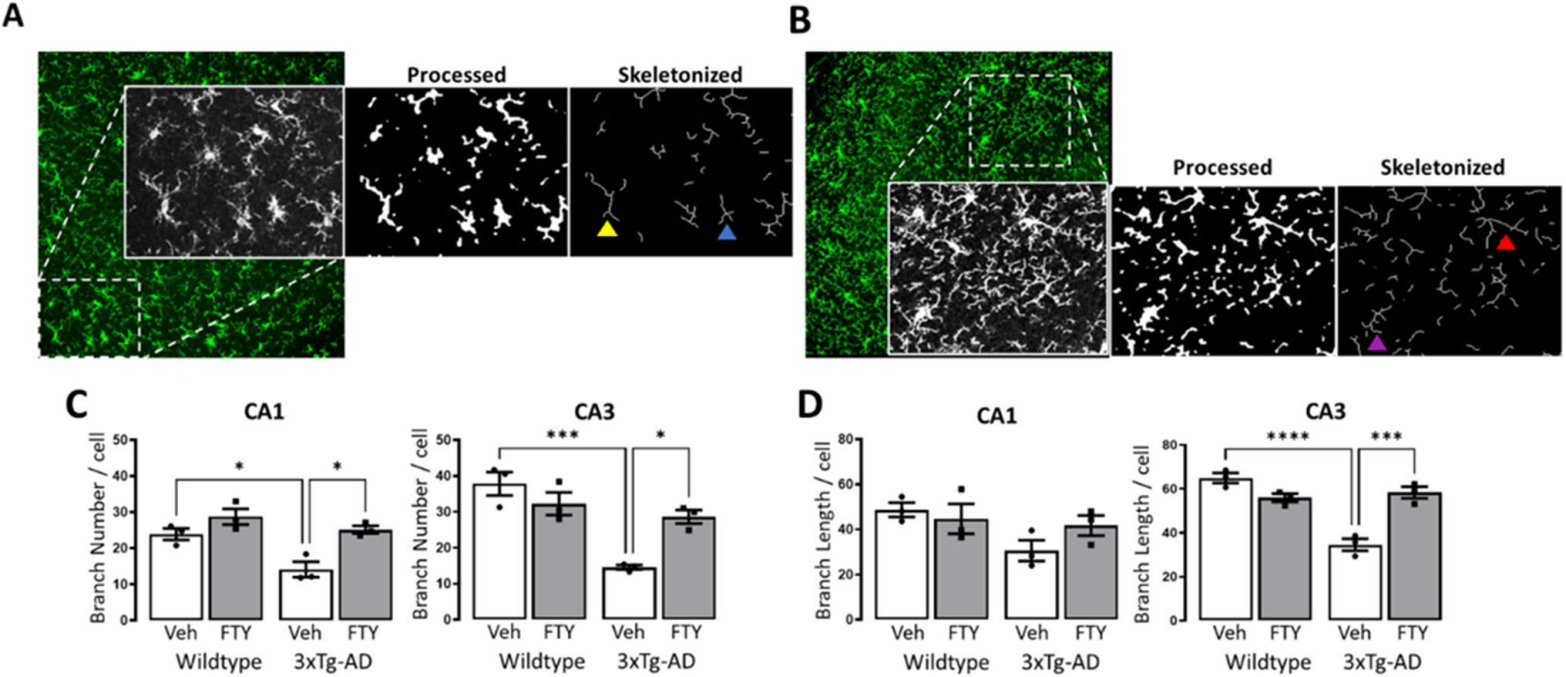
Fingolimod reduces microglia reactivity in 3xTg-AD mice. Iba1-positive cells were assessed by skeleton analysis to determine (**A**) activated or (**B**) ramified morphology through branch number (low branch number yellow arrow, high branch number purple arrow) and branch length (short branch length blue arrow, long branch length red arrow). **C** A genotype-related reduction in branch number was observed in the CA1 (*p* < 0.0061, *F*_(1,8)_ = 13.65) and CA3 (*p* = 0.0006, *F*_(1,8)_ = 29.49). Post hoc analysis revealed that microglia in vehicle treated 3xTg-AD mice had significantly fewer branches than in WT (CA1: *p* = 0.0215, *n* = 3; CA3: *p* = 0.007, *n* = 3) and that this was reversed with fingolimod treatment (CA1: *p* = 0.0111, *n* = 3; CA3: *p* = 0.0163, *n* = 3). **D** A genotype-treatment interaction in microglial branch length was observed in the CA3 (*p* = 0.0001, *F*_(1,8)_ = 45.61) but not CA1. Post hoc analysis revealed that branch length in vehicle treated 3xTg-AD mice was smaller than that in WT controls (*p* < 0.0001, *n* = 3) and that increased branch length was observed in fingolimod treated 3xTg-AD mice (*p* = 0.0005, *n* = 3). Data are expressed as the mean ± SEM, *p < 0.05, ***p < 0.001, ****p < 0.0001

Inflammation in the CNS compounds the progression of AD and is an important therapeutic target [41]. We therefore investigated the functional state of microglia in fingolimod-treated 3xTg-AD mice by assessing the proliferation (Fig. 3A, C) and morphology (Fig. 4A, B) of microglia in the hippocampus. A genotype-related increase in the number of Iba1-positive cells was observed in the CA1 (*p* = 0.0286, *F*_(1,8)_ = 7.098; Fig. 3B) and CA3 (*p* = 0.0076, *F*_(1,8)_ = 12.56; Fig. 3D) and post hoc analysis revealed that treatment with fingolimod significantly reduced the number of Iba1-positive cells in 3xTg-AD mice (Veh CA1: 0.251 ± 0.006, FTY CA1: 0.201 ± 0.009, *p* = 0.0174, *n* = 3; Veh CA3: 0.233 ± 0.012, FTY CA3: 0.169 ± 0.013; *p* = 0.0151, *n* = 3). Skeleton analysis was carried out to further examine the morphology of microglia. A genotype-related reduction in branch number was observed in the CA1 (*p* = 0.0061, *F*_(1,8)_ = 13.65; Fig. 4C) while branch number (*p* < 0.0006, *F*_(1,8)_ = 29.49; Fig. 4C) and length (*p* = 0.0001, *F*_(1,8)_ = 45.61; Fig. 4D) was reduced in the CA3. Post hoc analysis determined that treatment with fingolimod increased microglial branch number in the CA1 (Veh: 14.13 ± 2.13, FTY: 25.15 ± 1.07; *p* = 0.0111, *n* = 3) and CA3 (Veh: 14.57 ± 0.63, FTY: 28.64 ± 1.88; *p* = 0.0163, *n* = 3) as well as increasing microglial branch length in the CA3 (Veh: 34.6 ± 2.75, FTY: 58.37 ± 2.69; *p* = 0.0005) of 3xTg-AD mice. Taken together these data reveal that 3xTg-AD mice display increased microglial proliferation and the development of a reactive microglial morphology and that treatment with fingolimod reduces this microglial reactivity.

### Fingolimod Reduces Circulating and Infiltrating Lymphocytes

**Fig. 5:**
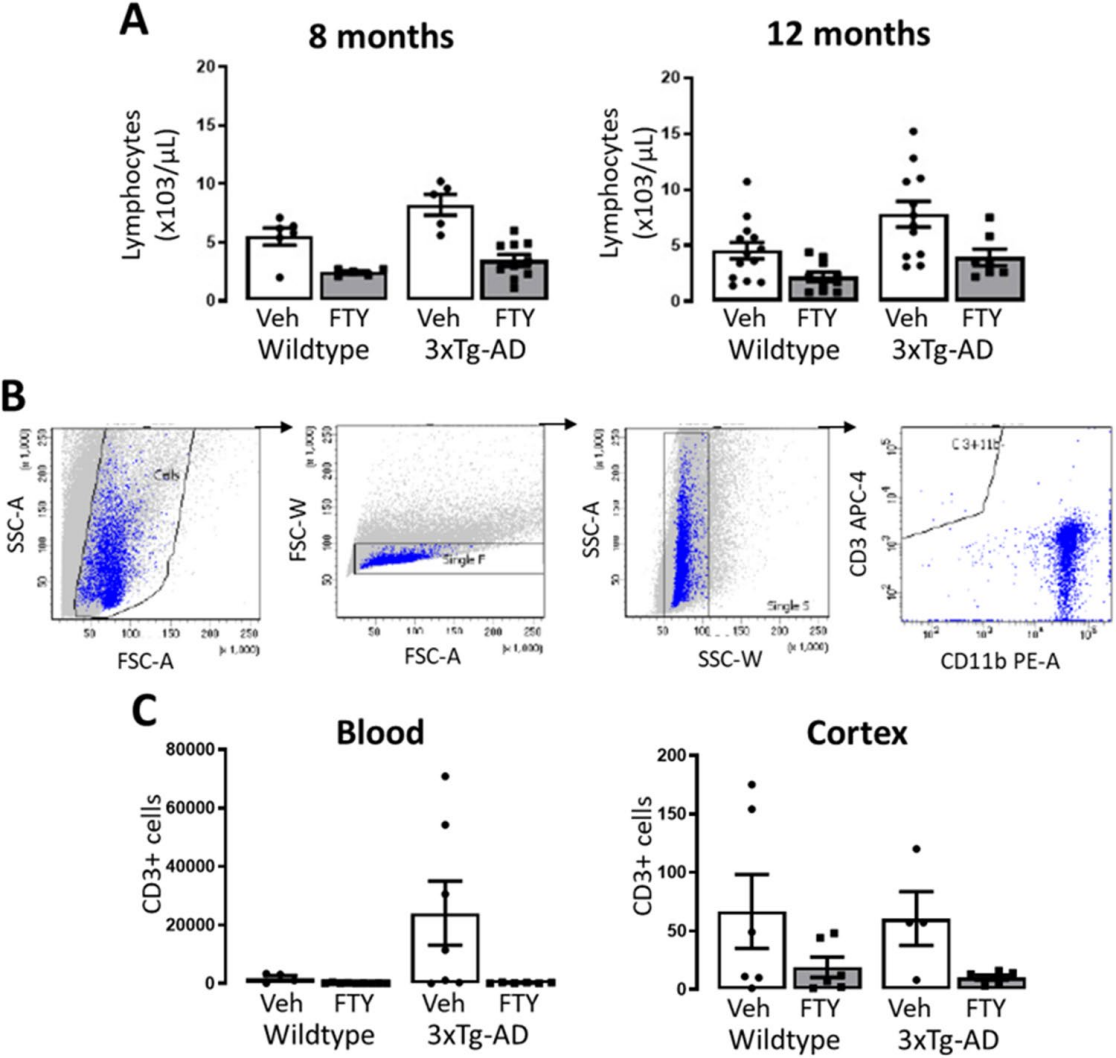
Fingolimod reduces circulating and CNS-infiltrating lymphocytes. **A** Blood was drawn from the sub-mandibular vein and lymphocytes measured on the Sysmex KX-21. A significant genotype-related increase in circulating lymphocytes was observed at 8- (*p* = 0.0079, *F*_(1,22)_ = 8.524, *n* = 5–10) and 12 months (*p* = 0.0077, *F*_(1,38)_ = 7.917, *n* = 7–12) of age. Concomitantly a treatment-related reduction was also observed at 8 (*p* < 0.0001, F_(1,22)_ = 36.49, *n* = 5–10) and 12 months (*p* = 0.0013, *F*_(1,38)_ = 12.07, *n* = 7–12) of age. **B** Representative gating strategy for sorting CD3 + T cells. **C** FACS analysis confirmed the treatment-related reduction in circulating CD3 + T cells (*p* = 0.049, *F*_(1,22)_ = 4.335, *n* = 6–9) and CD3 + T cells in the cortex (*p* = 0.026, *F*_(1,18)_ = 5.925, *n* = 4–6). Data are expressed as the mean ± SEM

The canonical activity of fingolimod is well described and involves the retention of lymphocytes in lymph nodes [10]. As expected, a treatment-related reduction in circulating lymphocytes was detected in mice treated with fingolimod after 2 (*p* < 0.0001, *F*_(1,22)_ = 36.49) and 6 (*p* = 0.0013, *F*_(1,38)_ = 12.07) months of treatment (Fig. 5A). FACS analysis was carried out on blood and cortical tissue from experimental animals at 12 months of age (Fig. 5B). Cell sorting confirmed the treatment-related reduction in circulating CD3 + T cells (*p* = 0.049, *F*_(1,22)_ = 4.335; Fig. 5C) and also demonstrated that fingolimod reduced the number of infiltrating CD3 + T cells in the cortex (*p* = 0.026, *F*_(1,18)_ = 5.925; Fig. 5C).

### Fingolimod Improves the Cytokine Profile in 3xTg-AD Mice

**Fig. 6:**
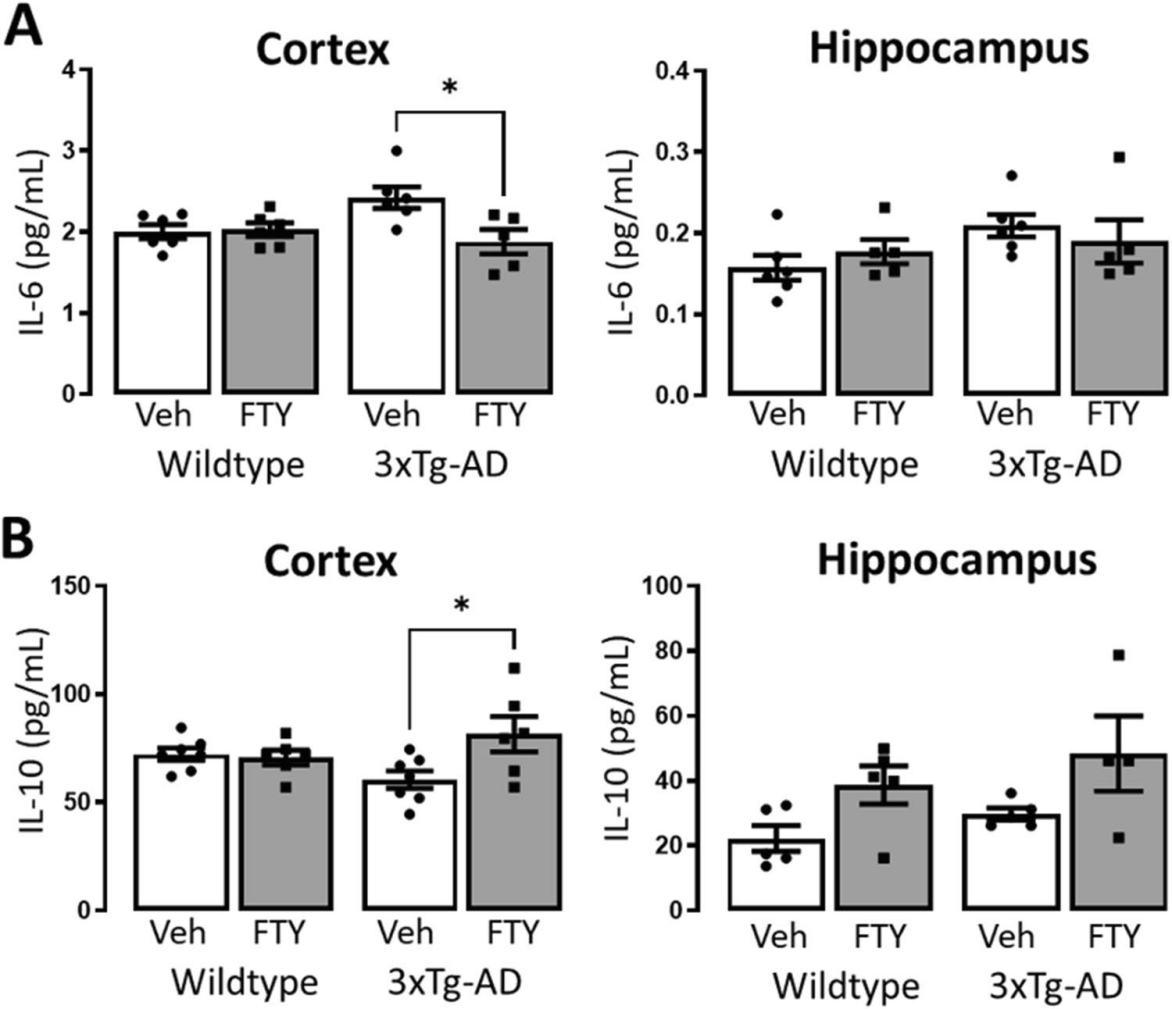
Fingolimod alters the cytokine profile in the 3xTg-AD brain. IL-6 and IL-10 concentrations in the cortex and hippocampus were assessed by ELISA. **A** A genotype-treatment interaction in IL-6 was observed in the cortex (*p* = 0.0225, *F*_1,19_ = 6.169) but not hippocampus. Post hoc analysis revealed a significant reduction in fingolimod-treated 3xTg-AD mice compared to vehicle treated (*p* = 0.0182, *n* = 5–6). **B** A significant genotype-treatment interaction was observed in IL-10 levels in the cortex (*p* = 0.0298, *F*_(1,22)_ = 5.398) and a treatment-related increase in IL-10 was observed in the hippocampus (*p* = 0.0121, *F*_(1,15)_ = 8.138). Post-hoc analysis revealed that 3xTg-AD mice treated with fingolimod had significantly higher levels of IL-10 in the cortex compared with vehicle controls (*p* = 0.0277, *n* = 6–7). Data are expressed as the mean ± SEM, *p < 0.05

We next quantified the levels of the pro- and anti-inflammatory cytokines, IL-6 and IL-10, in tissue homogenates of the hippocampus and cortex using ELISA. No difference in levels of hippocampal IL-6 was observed between groups; however, a genotype/treatment interaction was observed in cortical IL-6 (*p* = 0.0225, *F*_(1,19)_ = 6.169; Fig. 6A). Post hoc analysis determined that cortical IL-6 was significantly reduced in 3xTg-AD mice receiving fingolimod compared to vehicle controls (Veh: 2.424 ± 0.13, FTY: 1.881 ± 0.15; *p* = 0.0182, *n* = 5–6). A treatment-related increase in hippocampal IL-10 was observed (*p* = 0.0121, *F*_(1,15)_ = 8.138; Fig. 6B). In cortical tissue, a genotype/treatment interaction was observed (*p* = 0.0298, *F*_(1,22)_ = 5.398; Fig. 6B) and post hoc analysis revealed that IL-10 was significantly increased in 3xTg-AD mice receiving fingolimod compared to vehicle-treated controls (Veh: 60.57 ± 4.04, FTY: 81.58 ± 8.15; *p* = 0.0277, *n* = 6–7). These data support a shift in the cytokine profile of 3xTg-AD mice after fingolimod treatment from pro-inflammatory to anti-inflammatory.

### Fingolimod Reduces Tau Phosphorylation and APP Expression in 3xTg-AD Mice

**Fig. 7:**
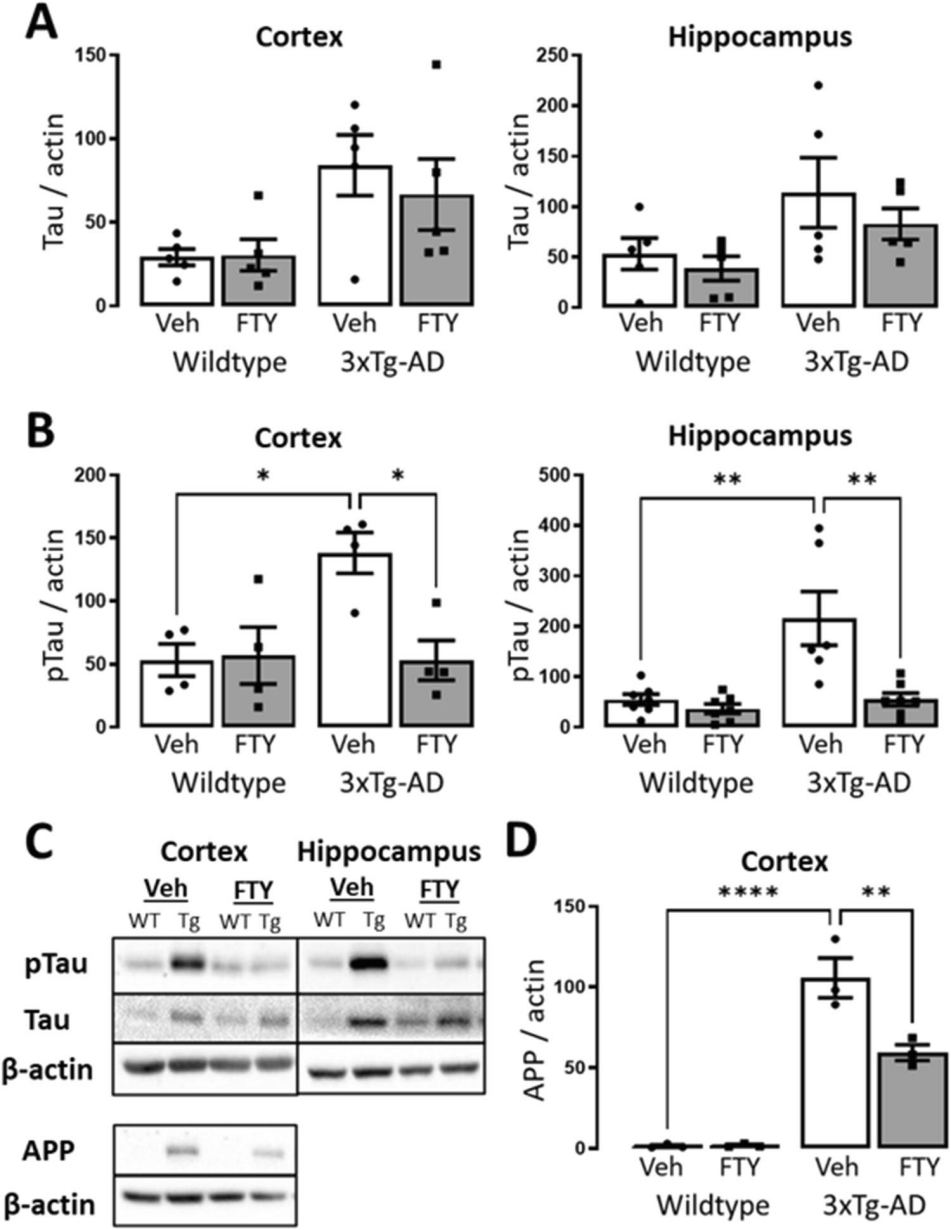
Fingolimod reduces pathological hallmarks in 3xTg-AD mice. **C** The protein levels of Tau (Tau46), phospho-Tau (AT8), and APP (4G8) were measured in the cortical and hippocampal tissue of experimental mice. **A** A genotype-related increase in tau protein was observed in the cortex (*p* = 0.0076, *F*_(1,16)_ = 9.306) and hippocampus (*p* = 0.0265, *F*_(1,16)_ = 5.975). **B** A concomitant genotype-related increase in phospho-tau protein was also observed in the cortex (*p* = 0.0242, *F*_(1,12)_ = 6.649) and hippocampus (*p* = 0.0018, *F*_(1,23)_ = 12.5). Post hoc analyses revealed that phospho-tau protein was increased in 3xTg-AD mice compared to WT (cortex: *p* = 0.02, *n* = 4; hippocampus: *p* = 0.0011, *n* = 6–7) and that treatment with fingolimod alleviated this (cortex: *p* = 0.0198, *n* = 4; hippocampus: *p* = 0.0012, n = 6–7). **D** A significant genotype-related increase was observed in the level of cortical APP (*p* < 0.0001, *F*_(1,8)_ = 147.6). Post hoc analysis revealed that APP was increased in 3xTg-AD mice compared to WT (*p* < 0.0001, *n* = 3) and that this was reduced by treatment with fingolimod (*p* = 0.0051, *n* = 3). Data are expressed as the mean ± SEM, *p < 0.05, **p < 0.01, ****p < 0.0001

Neurofibrillary tangles of hyperphosphorylated tau and amyloid plaques are hallmarks of AD and develop in 3xTg-AD [16, 59]. Phospho-tau burden and APP load were assessed through immunoblot (Fig. 7C). As expected a significant genotype-related increase in total tau protein was observed in the cortex and hippocampus of 3xTg-AD mice by Tau46 antibody (cortex: *p* = 0.0076, *F*_(1,16)_ = 9.306; hippocampus: *p* = 0.0265, *F*_(1,16)_ = 5.975; Fig. 7A). A concomitant genotype-related increase in tau phosphorylated at Ser202/Thr205 was observed using AT8 antibody (cortex: *p* = 0.0242, *F*_(1,12)_ = 6.649; hippocampus: *p* = 0.0018, *F*_(1,23)_ = 12.5; Fig. 7B). Post hoc analysis revealed that treatment with fingolimod significantly reduced the level of phosphorylated tau in the cortex (Veh: 138.2 ± 16.23, FTY: 53.15 ± 15.82; *p* = 0.0198, *n* = 4) and hippocampus (Veh: 215.9 ± 53.16, FTY: 55.44 ± 12.27; *p* = 0.0012, *n* = 6–7) of 3xTg-AD mice. The deposition of A*β* plaques is another key feature in the development of AD, and while extensive plaque burden is present in female 3xTg-AD mice previous reports have demonstrated limited plaque burden in 3xTg-AD males of this age [13]. Consistent with this, we observed a limited plaque burden in our mice (Supp. Figure 2). We then measure the level of the A*β* parent protein, APP. A genotype-related increase in APP was observed in the cortex of 3xTg-AD mice using 6E10 antibody (*p* < 0.0001, *F*_(1,8)_ = 147.6; Fig. 7D). Post hoc analysis determined that treatment with fingolimod reduced the level of APP in the cortex of 3xTg-AD mice (Veh: 105.7 ± 12.29, FTY: 59.38 ± 4.99; *p* = 0.0051, *n* = 3). These data confirm the pathological phosphorylation of tau at Ser202/Thr205 in 3xTg-AD mice and reveal that fingolimod treatment reduces both APP expression and tau phosphorylation.

## Discussion

In this study, we investigated the effect of fingolimod, an S1P receptor antagonist, on AD phenotypes in the 3xTg-AD model. Animals were aged for 6 months to allow for the development of pathology and fingolimod was then administered for a further 6 months via the drinking water. The data demonstrate that, in aged 3xTg-AD mice, treatment with fingolimod reverses disease-associated working memory impairment, alleviates inflammation in the CNS, and reduces the levels of phosphorylated tau and APP.

AD is characterized by the progressive decline in cognition, where memory impairment and behavioral disturbances affect daily activities [23, 26]. In 3xTg-AD mice impaired spatial working memory and increased anxiety have been reported from 3 and 6 months of age, respectively [16, 18, 67, 80]. Previous studies have highlighted the potential benefits of fingolimod administration on memory impairment, however, in those studies treatment began prior to the decline in cognition and must be considered a preventative measure [6, 12, 32]. Our data demonstrate that administration of fingolimod after the reported onset of memory decline in 3xTg-AD mice results in a restoration of spatial working memory extending to 12 months of age.

Inflammation in the CNS is an important factor in the onset and progression of AD. Recent studies using positron emission tomography have revealed chronic microglial activation in the AD brain and have used this as a predictor of cognitive decline [46–48, 61]. Postmortem analyses have also confirmed the significant increase in microglial activation observed in the AD brain [36, 70]. In 3xTg-AD mice increased microglial number and elevated proinflammatory cytokines have been reported from 6 months of age [12, 14, 32, 37]. In line with this, we report increased microglial number in the hippocampus of 12-month 3xTg-AD mice compared to WT controls and have demonstrated that these cells develop an activated morphology as measured by reduced branch length and number. Consistent with previous studies we have shown that treatment with fingolimod reduces the number of microglial cells and, furthermore, have demonstrated that microglia in treated mice display a resting morphology.

Mounting evidence has highlighted the infiltration of peripheral lymphocytes to the brain parenchyma of AD patients [24, 51, 75]. This has also been reported in APP and tau transgenic mice and has been shown to further compound inflammation in the CNS [11, 25, 43]. Here, we report for the first time the detection of CD3 + T-cell lymphocytes in the cortex of 3xTg-AD mice. Furthermore, treatment with fingolimod reduced the level of circulating lymphocytes and we note a concomitant reduction in CD3 + T-cell lymphocytes in the brains of 3xTg-AD mice.

Previous A*β*-infusion models of AD have shown that fingolimod reduces proinflammatory TNF-*α* and COX-II levels while increasing brain-derived neurotrophic factor (BDNF) [6],Fukumoto et al. 2014a; [32]. Similarly, our data have identified a fingolimod-mediated switch from a pro-inflammatory to anti-inflammatory cytokine profile in the cortex and hippocampus of 3xTg-AD mice with a reduction in IL-6 and an increase in IL-10 concentrations. Interestingly, the effect of fingolimod on IL-6 and IL-10 is observed in 3xTg-AD mice only and not WT. No genotype-related alteration in these cytokines was recorded. Further research is required to elucidate this genotype-specific effect of fingolimod.

The characteristic hallmarks of AD are the deposition of extracellular amyloid plaques and the formation of intraneuronal neurofibrillary tangles of hyperphosphorylated tau [21]. These pathologies have been implicated in the death of the neuronal population and chronic activation of the immune response [17, 34, 42, 45]. In 3xTg-AD mice both amyloid and tau pathology develop by 6 and 12 months of age, respectively [59]. Previous in vitro and in vivo studies have demonstrated that fingolimod reduces A*β* production in neurons and the formation of amyloid plaques [7, 72], and reduces APP expression in Schwann cells [2]. The use of male mice only in this study limited our examination of A*β*40/42 levels and plaque burden [13], and we note that future studies should include the examination of female mice in the cohort. However, consistent with previous findings, our data indicates that fingolimod reduces the expression of APP in the cortex of 3xTg-AD mice which may explain the reduction in A*β* previously reported. Furthermore, alteration of the S1P system in the form of reduced S1P-lyase has been shown to affect APP degradation [40]. While out of the scope of this current study the effect of the S1P system on APP production and degradation merits further study. Finally, we demonstrate for the first time that fingolimod reduces the level of phosphorylated tau in the cortex and hippocampus of 3xTg-AD mice with no effect on total tau. One possible mechanism is through the reduction in glycogen synthase 3*β*, one of the primary kinases for tau. While it has not yet been demonstrated in neurons, fingolimod has been shown to reduce the level of glycogen synthase 3*β* in a number of cell types [44, 64, 74].

AD is a multifactorial disease involving the complex interaction between amyloid plaque formation, phosphorylated tau misfolding, and chronic inflammation in the CNS resulting in widespread neuronal loss and fatal cognitive decline. Therapeutic intervention is currently limited and clinical trials targeting these hallmarks individually have yielded little success. This study has demonstrated that treatment with fingolimod reduces inflammation in the CNS, reduces APP and tau phosphorylation, and restores spatial working memory in male 3xTg-AD mice. The results here add to the increasing body of evidence indicating that fingolimod should be considered a viable therapeutic agent for the treatment of AD.

## Supplementary Information

Below is the link to the electronic supplementary material.

Supplementary file1 (DOCX 1926 kb)

## Data Availability

The datasets generated during and/or analyzed during the current study are available from the corresponding author on reasonable request.

## Abbreviations

AD: Alzheimer’s disease
A*β*: Amyloid-beta
APP: Amyloid precursor protein
FTY720: Fingolimod, Gilenya™
S1P: Spingosine-1-phosphate
CNS: Central nervous system
3xTg-AD: B6;1239-Tg(APPSwe,tauP301L)1Lfa Psen1^tm1Mpm^ mice
WT: Wild-type mice
NOL: Novel object location
MWM: Morris water maze
