# Supplementary material for: Fingolimod Rescues Memory and Improves Pathological Hallmarks in the 3xTg-AD Model of Alzheimer’s Disease
Source: Mol Neurobiol. 2022 Jan 15;59(3):1882–95. doi: 10.1007/s12035-021-02613-5 (PMC8882098; doi:10.1007/s12035-021-02613-5)
Supplement: Supplementary file 1 — Supplementary file1 (DOCX 1926 kb) [file 12035_2021_2613_MOESM1_ESM.docx]

**Supplementary Information for manuscript:**

**Fingolimod rescues memory and improves pathological hallmarks in the 3xTg-AD model of Alzheimer’s disease.**

Steven G. Fagan, Sibylle Bechet and Kumlesh K. Dev

Drug Development, School of Medicine, Trinity College Dublin, Ireland.

**
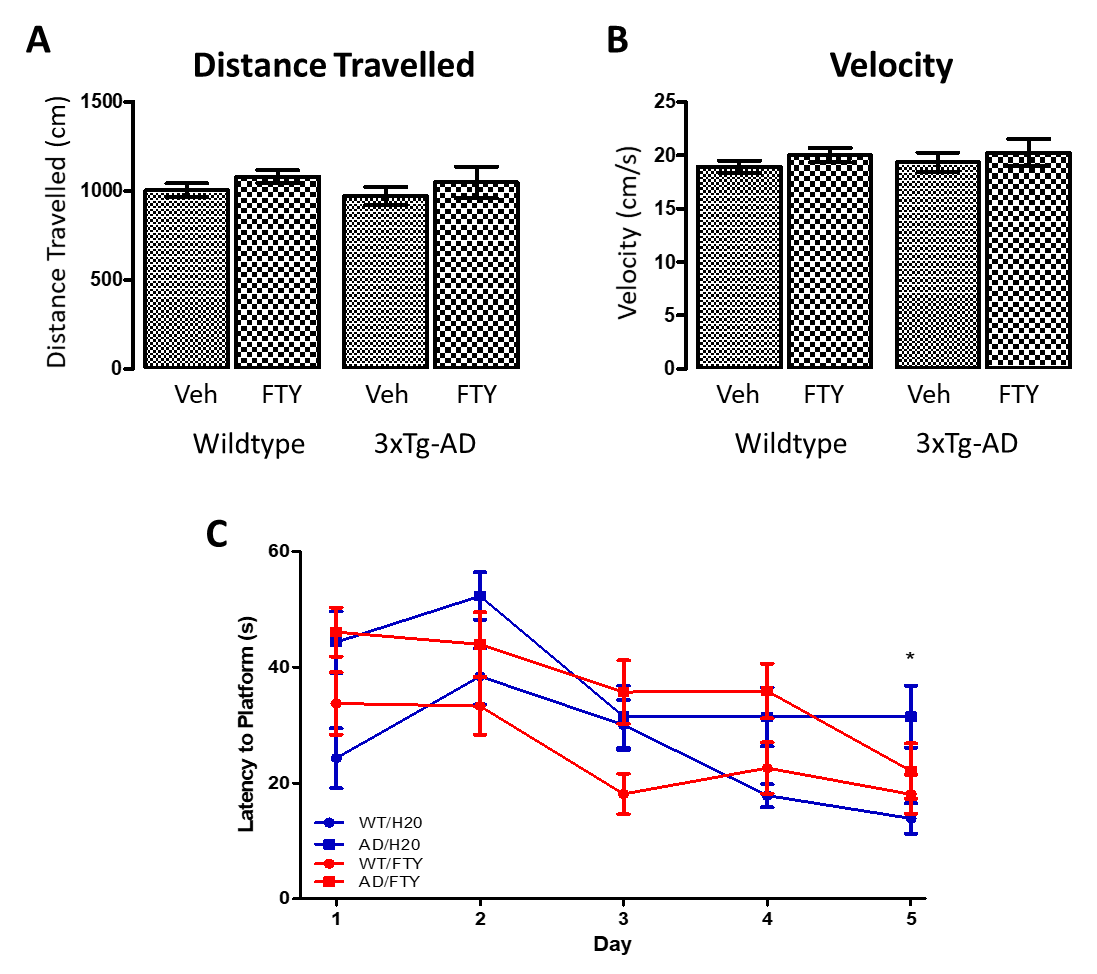
**

**Supplementary Figure 1:** No difference in athletic ability was observed in mice undertaking the Morris water maze test as indicated by (A) total distance travelled, and (B) velocity. (C) The escape latency of groups over acquisition and training days 1 – 5. On training day 5 vehicle treated 3xTg-AD mice perform significantly worse than WT counterparts (p=0.017, n=14-18).

**
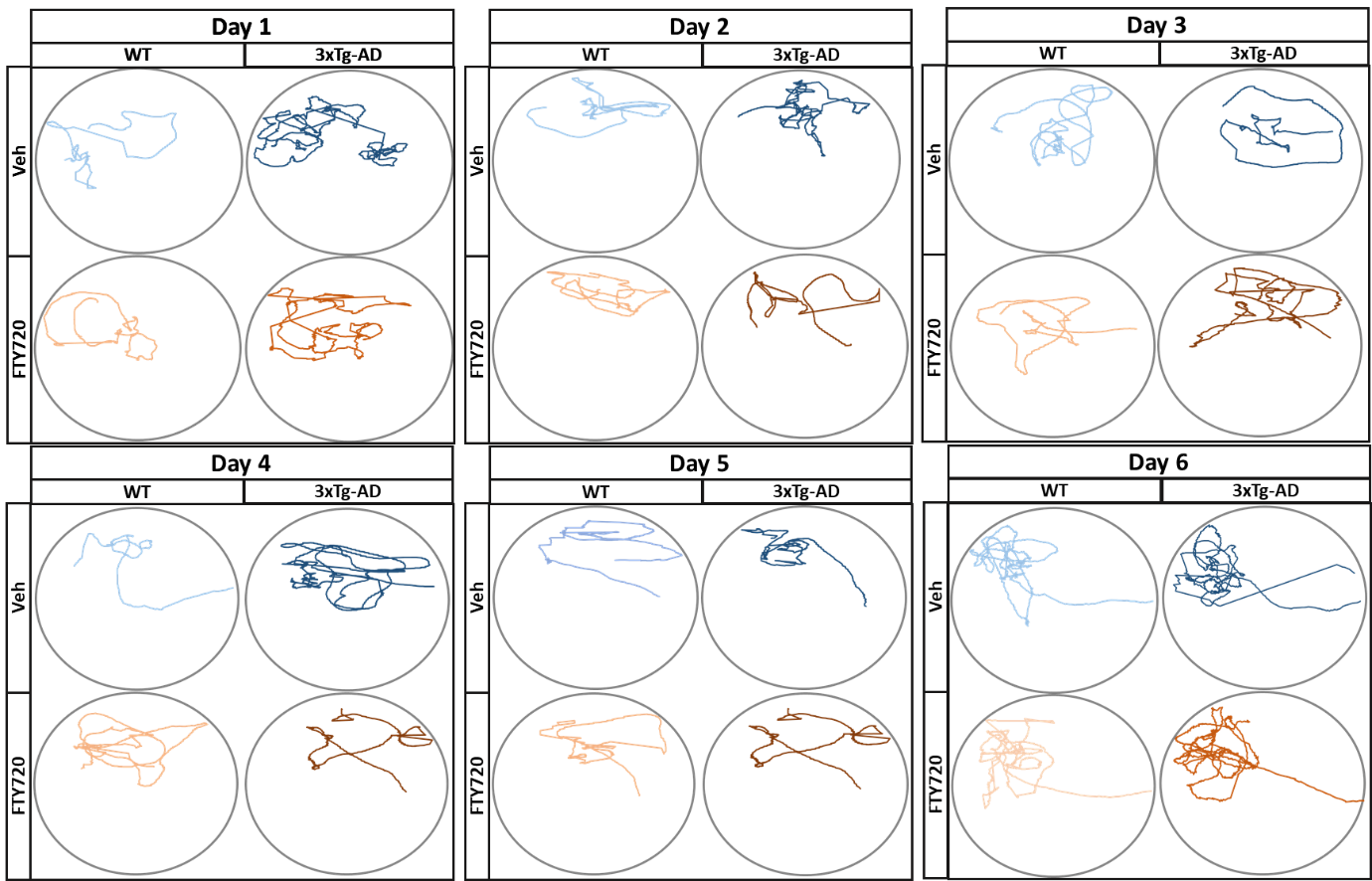
**

**Supplementary Figure 2:** Average swimming traces from Morris water maze of mice on days 1 (habituation), 2 – 5 (training) and 6 (test).

**
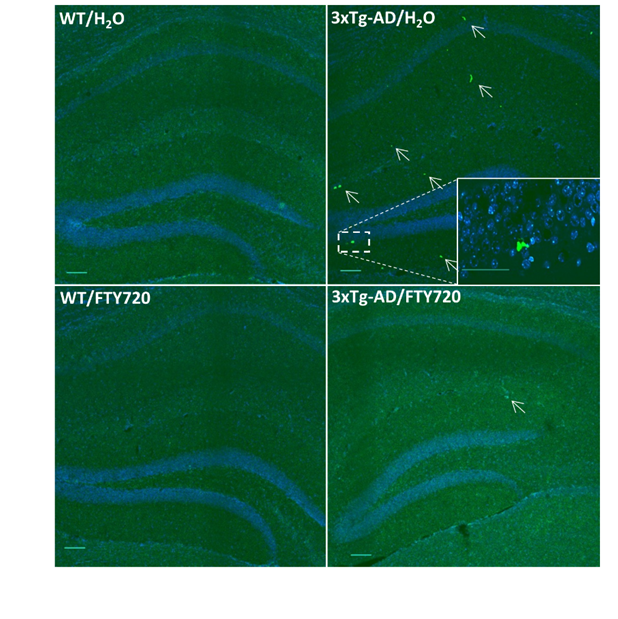
**

**Supplementary Figure 3:** Immunohistochemical labelling of Aβ plaques in the hippocampus of experimental animals using 6E10 antibody (1:500; Biolegend, SIG39320). Scale bar 100 µm.

**
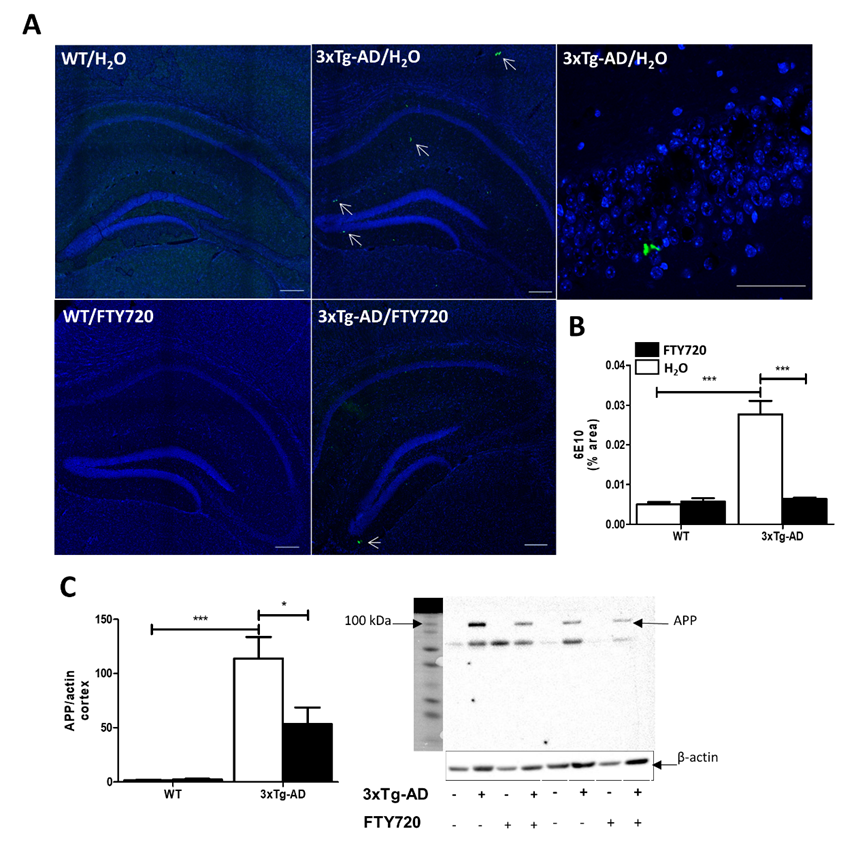
**

**Supplementary Figure 3:** Full western immunoblot of APP on cortical tissue samples from experimental groups.
